# Phosphorylation-Dependent SUMOylation of the Transcription Factor NF-E2

**DOI:** 10.1371/journal.pone.0044608

**Published:** 2012-09-10

**Authors:** Yee-Fun Su, Yu-Chiau Shyu, Che-Kun James Shen, Jaulang Hwang

**Affiliations:** 1 Institute of Molecular Biology, Academia Sinica, Taipei, Taiwan; 2 Department of Biochemistry, Medical School, Taipei Medical University, Taipei, Taiwan; 3 Institute of Biopharmaceutical Sciences, National Yang-Ming University, Taipei, Taiwan; 4 Department of Education and Research, Taipei City Hospital, Taipei, Taiwan; Chang Gung University, Taiwan

## Abstract

Nuclear factor erythroid-derived 2 (NF-E2), a heterodimer composed of p45 and p18, is a transcriptional activator in hematopoietic progenitors. The transcriptional activity of NF-E2 is not only upregulated by SUMOylation but also stimulated by the cAMP-dependent protein kinase A (PKA). However, the relationship between SUMOylation and phosphorylation in the activation of NF-E2 is unclear. In the present studies, we have demonstrated that PKA enhances NF-E2 SUMOylation in an *in vitro* system using purified proteins, suggesting a possible mechanism for PKA-dependent activation of the NF-E2 transcription factor through SUMOylation.

## Introduction

Nuclear factor erythroid-derived 2 (NF-E2), a heterodimer comprised of a large tissue-specific p45 subunit and a small p18 Maf family subunit, plays an essential role in regulating multiple erythroid- and megakaryocytic-specific genes [1], [2], [3]. Murine erythroleukemia (MEL) cells lacking p45 show a reduction in globin gene expression, [4]. Mice lacking NF-E2 exhibit thrombocytopenia accompanied by defects in platelet production [5]. Patients with polycythemia vera disease (PV) present with elevated concentration of p45/NF-E2, overproduction of red cell and thrombocytopenia [6]. Thus, tight control of NF-E2 activity is essential during erythroid differentiation.

Post-translational modification has been shown to regulate NF-E2 transcriptional activity. cAMP-dependent protein kinase A (PKA) increases NF-E2/DNA complex formation in Murine erytholeukemia cell differentiation [7]. The Ras signaling cascade has also been shown to be promote the transactivational function of NF-E2 [8]. Additionally, small ubiquitin-like modifiers (SUMO) have been shown to exert control over NF-E2 transcription [9].

SUMOylation is a multi-step enzymatic modification that begins with the activation and attachment of a SUMO protein to an E1-activating enzyme (SAE1/SAE2). The SUMO moiety is then transferred from the E1 to the E2-conjugating enzyme (Ubc9) and finally transferred to the lysine residues of a target protein, which is enhanced by E3 ligases [10]. SUMOylation participates in a broad range of biological functions including transcription factor activity, subcellular localization and cell cycle regulation. In transcriptional regulation, SUMOs mediate transcriptional activities of activators, coactivators, repressors, corepressors and other subnuclear structures [11]. SUMOylation also exerts negative regulation over transcriptional activity. For example, the transcriptional activities of Sp3, c-Myb and p300 are repressed as a consequence of SUMOylation [12], [13], [14]. Also, the transcriptional activity of p45/NF-E2 is enhanced by SUMO modification [9], while the SUMOylation of its obligatory binding partner, p18/MafG, results in transcriptional repression by the recruitment of histone deacetylase [15].

The effects of the interplay between phosphorylation and SUMOylation have been widely studied in recent years [16]. The presence of a phosphorylation-dependent SUMOylation motif (PDSM) seen in several SUMO target proteins suggests an important relationship between phosphorylation and SUMOylation [17], [18]. Phosphorylation within the PDSM motif may positively regulate the SUMOylation activity of a substrate protein, as is seen in the enhancement of SUMOylation activities by phosphorylation of the heat shock factors (HSFs) and myocyte enhancing factor-2 (MEF2) family members [18], [19], [20], [21]. By contrast, phosphorylation may also negatively regulate the SUMOylation activity of substrate proteins. Phosphorylation has been shown to reduce the SUMOylation of the ELK1, c-Fos and c-Jun transcription factors [22], [23], [24]. Since the transcriptional activity of NF-E2 is upregulated by both phosphorylation (e.g. by PKA) and SUMOylation, it is possible that there may be an interplay between PKA-dependent phosphorylation and SUMOylation in the regulation of the NF-E2 transcriptional activity.

In order to investigate the possible interplay between PKA-dependent phosphorylation and SUMOylation, we have performed *in vitro* experiments using purified proteins. Our results demonstrate that PKA enhances SUMOylation of NF-E2, suggesting an interplay between phosphorylation and SUMOylation in the activation of NF-E2 transcriptional activity.

## Results

### Expression and Purification of p45/NF-E2 Heterodimer and p45 Monomer

**Figure 1:**
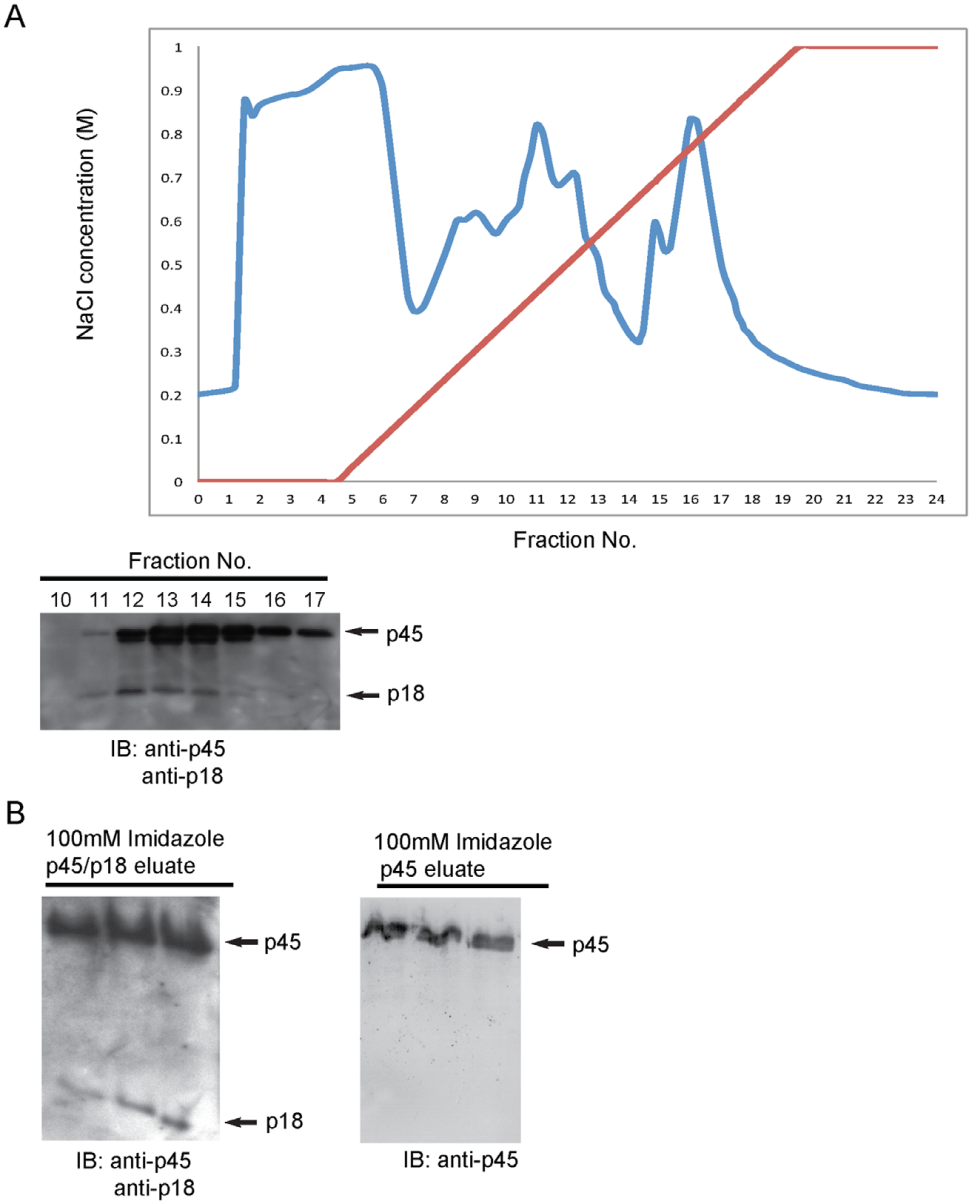
Expression and purification of p45/NF-E2 heterodimer and p45. A) Whole cell lysates of baculovirus co-expressing p45 and p18 were subjected to Mono Q Sepharose anion-exchange chromatography. Proteins were eluted by a NaCl gradient in 50 mM Tris-HCl, pH 8.0 buffer. All isolated fractions (#1–24) were collected by FPLC. The fractions (#11–14) primarily contain the p45/p18 heterodimer. Fractions (#10–17) were subjected to Western blotting with antibody against p45 or p18. B) Eluates containing the p45/p18 heterodimer (fractions #11–14) were subjected to nickel affinity purification and proteins were eluted with 100 mM imidazole. Eluates containing the p45 monomer were similarly purified by nickel affinity column. The purified p45/p18 heterodimer and p45 monomer were resolved by 11.25% and 12.5% SDS-PAGE, respectively, and subsequently immunoblotted with p45 or p18 antibody as indicated.

Mono Q sepharose anion-exchange chromatography was used to isolate the p45/p18 heterodimer from SF21 cells co-expressing p45 and p18. p45 and p18 carry very different isoelectric points (pI), 4.8 vs. 10, respectively. By using the Mono Q Sepharose purification strategy, p45/p18 heterodimers and individual p45 and p18 molecules can be readily fractionated. As expected, p45 and p18 (representing the p45/p18 heterodimer) were co-eluted between 0.3 and 0.6 M NaCl (fractions #11–14). These fractions were subsequently pooled for further purification by nickel affinity chromatography (**Figure 1B** **; left panel**). To study SUMOylation of the p45 monomer in the absence of p18, the p45 monomer was expressed using the baculovirus expression system. The p45 monomer was eluted between 0.8 and 0.9 M NaCl (fraction # 16–17) using the Mono Q column (**Figure 1A** **; upper and lower left panels**). Fractions #16 and #17 were pooled and purified by nickel affinity chromatography (see Experimental Procedures) (**Figure 1B** **; right panel**).

### PKA Stimulates the SUMOylation of p45/p18 (NF-E2) Heterodimer in the Presence of ATP

**Figure 2:**
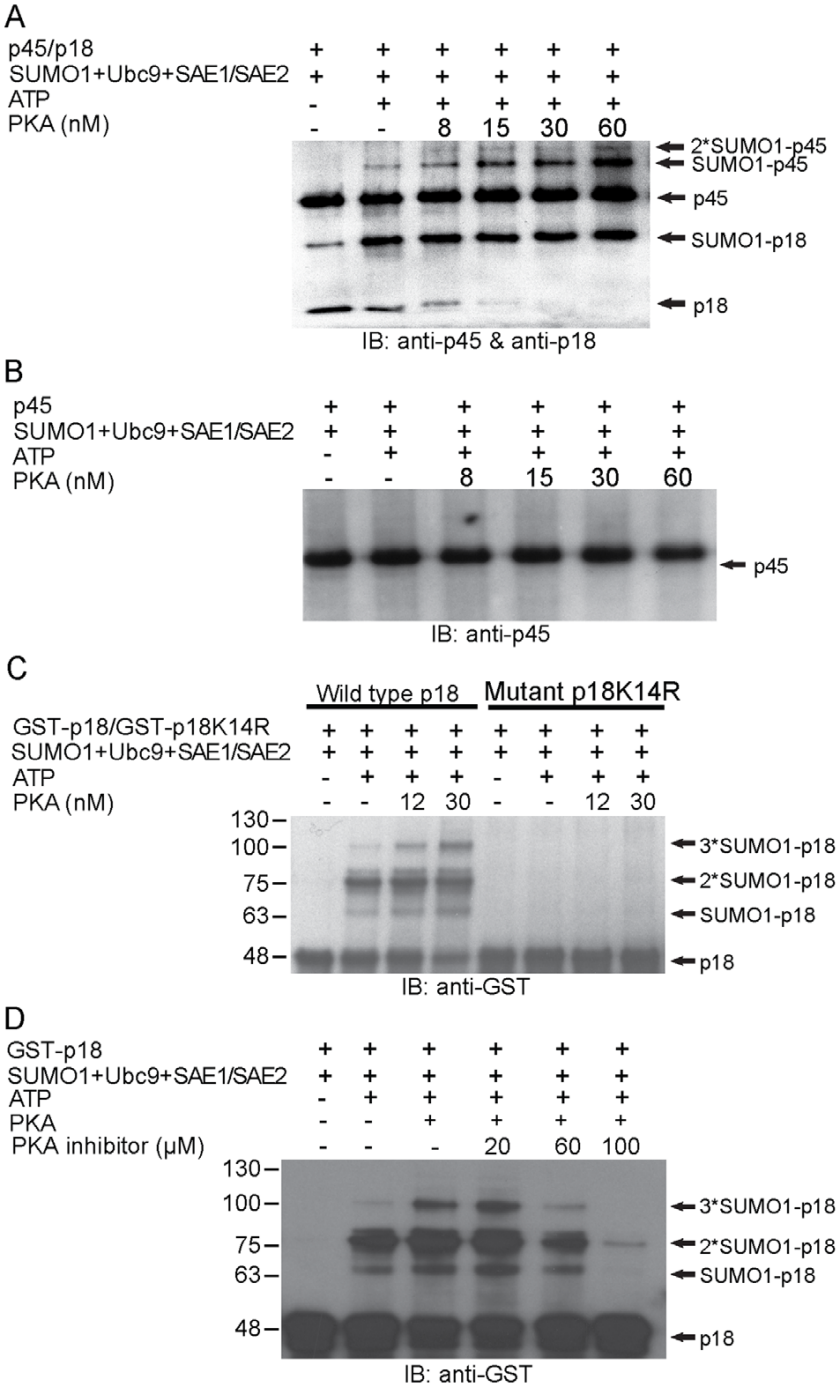
SUMOylation of p45/p18 heterodimer and p18, but not p45, is enhanced by PKA. A–B) Various concentrations (8 nM, 15 nM, 30 nM and 60 nM) of PKA were added in SUMOylation reaction mixtures containing His_6_-SUMO1, His_6_-Ubc9, SAE1/His_6_SAE2 (2.25 ng/µl), p45/p18 or p45 alone and ATP, which were then incubated at 37°C for 60 min. The reactions were then boiled in SDS sample buffer and resolved by 11.25% and 12.5% SDS-PAGE, followed by immunoblotting with anti-p45 or p18 antibody. C) GST-p18 or GST-p18K14R was added to the SUMOylation mixture in the increasing concentration (12 nM and 30 nM) of PKA and incubated at 37°C for 60 min. The reactions were subsequently boiled in SDS sample buffer, resolved by 10% SDS-PAGE, and immunoblotted with an anti-GST antibody. D) SUMOylation assay containing His_6_-SUMO1, His_6_-Ubc9, SAE1/His_6_SAE2 (2.25 ng/µl), GST-p18, PKA and ATP were mixed in the presence of various concentration (20 µM, 60 µM and 100 µM ) of PKA inhibitor (Promega) incubated at 37°C for 60 min. The reactions were then boiled in SDS sample buffer and separated by 10% SDS-PAGE, followed by immunoblotting with anti-GST antibody, respectively.

Several studies have shown that both phosphorylation and SUMOylation of the p45/p18 (NF-E2) heterodimer affect its transcriptional activity [7], [9]. More recent studies have demonstrated a phosphorylation-dependent SUMOylation motif (PDSM) residing within several substrate proteins. This report prompts us to examine whether phosphorylation may affect the function of NF-E2 either via upregulating or downregulating SUMOylation of NF-E2 and NF-E2 activity. In order to determine whether SUMOylation of NF-E2 is regulated by phosphorylation, we conducted *in vitro* SUMOylation of p45/NF-E2 in the presence of PKA and ATP using purified proteins (see Experimental Procedures). A very low level of SUMO1 conjugation of NF-E2 (on both p18 and p45 subunits) was observed in the absence of PKA (**Figure 2A**). However, the level of SUMO1-conjugated NF-E2 was greatly enhanced by PKA in a concentration-dependent manner (**Figure 2A**), suggesting that PKA-dependent phosphorylation of NF-E2 up-regulates NF-E2 SUMOylation. It was noted that SUMO1 conjugation of the p45 monomer was essentially undetectable either in the absence or presence of PKA (**Figure 2B**). It is unclear why SUMOylation of p45 requires the presence of p18 (i.e. the formation of the p45/p18 heterodimer). By contrast, SUMOylation level and extent of p18 is stimulated by PKA in a concentration-dependent manner (see bands marked by *2- and *3-SUMO1-p18) regardless of its physical association with p45 (**Figure 2C**). In addition, the enhanced p18 SUMOylation was significantly reduced by PKA inhibitor in a concentration-dependent manner (**Figure 2D** **)**, consistently indicating the NF-E2 SUMOylation-stimulatory effect of PKA.

### ERK1 also Stimulates SUMOylation of NF-E2

**Figure 3:**
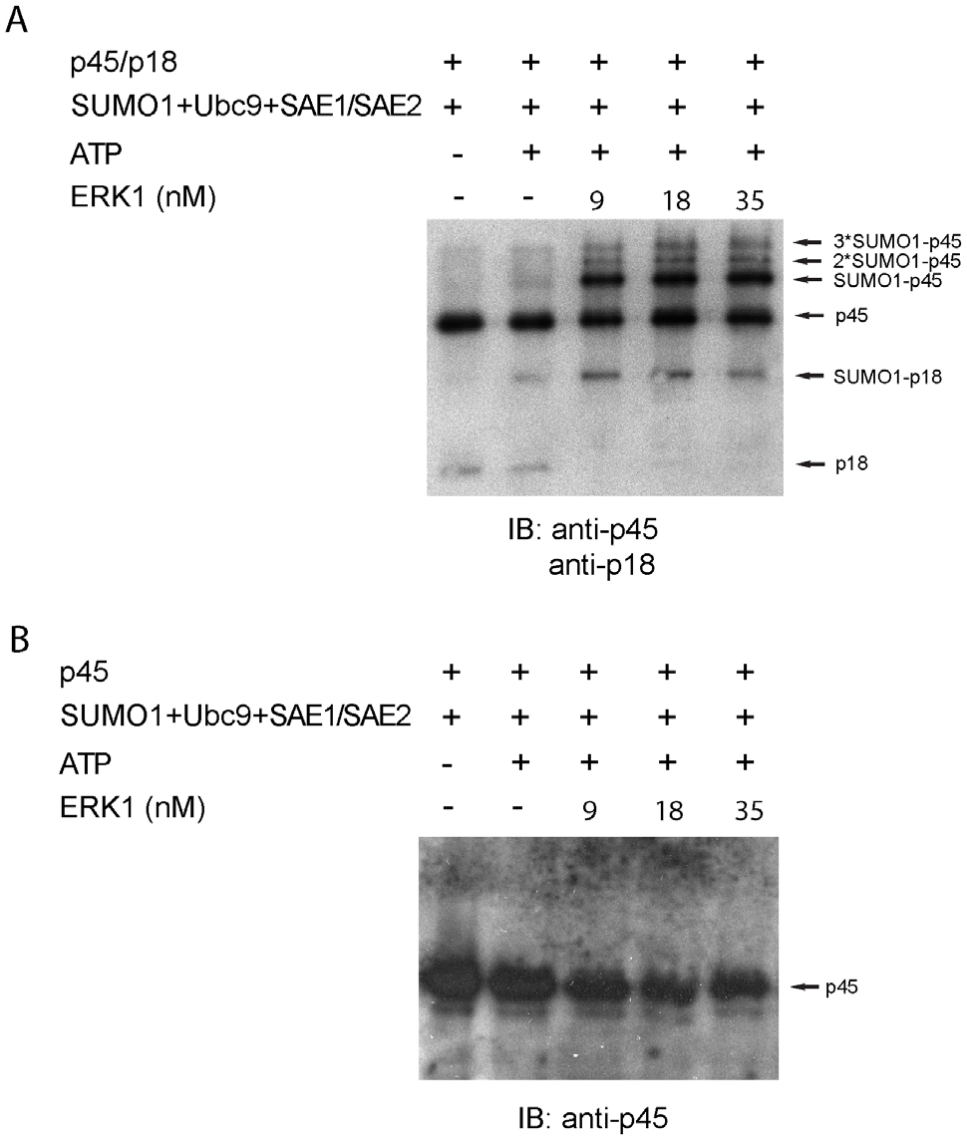
SUMOylation of p45/p18 heterodimer, but not p45, is enhanced by ERK1. A–B) Various concentrations (9 nM, 18 nM and 35 nM) of ERK1 were added to SUMOylation reaction mixtures containing His_6_-SUMO1, His_6_-Ubc9, SAE1/His_6_SAE2 (2.25 ng/µl), p45/p18 or p45 alone and ATP, which were then incubated at 37°C for 60 min. The reactions were then boiled in SDS sample buffer and separated by 11.25% and 12.5% SDS-PAGE, followed by immunoblotting with anti-p45 and p18 antibody, respectively.

Extracellular signal-regulated kinases (ERKs) are known to regulate a wide range of cellular physiological functions [25]. Previous studies have suggested that the DNA binding as well as the transactivation activity of NF-E2 in murine erythroleukemia cells (MEL) are regulated by the Ras signaling cascade [8]. Since ERK1 is one of the downstream kinases of the Ras signaling pathway, we examined whether ERK1 phosphorylation may also affect SUMOylation of NF-E2. We conducted *in vitro* SUMOylation of NF-E2 in the presence of ERK1 (see Experimental Procedures). In the absence of ERK1, only single SUMO1 (marked as SUMO1-p45) was conjugated to p45/NF-E2 at a low level (Fig. 3A). However, both the level and the extent (see bands marked by *2- and *3-SUMO1-p45) of SUMO1-conjugated p45/NF-E2 was enhanced by ERK1 in a concentration-dependent manner (**Figure 3A**), suggesting that ERK1 is likely to play a role in regulating SUMOylation of p45/NF-E2. By contrast, the SUMOylation level of the p45 monomer was undetectable either in the absence or the presence of ERK1, a result similar to that observed with PKA (**Figure 3B**) (see Discussion for a possible explanation). Similar to that of p45/NF-E2, SUMOylation of p18/NF-E2 was significantly increased in the presence of ERK1 (**Figure 3A**). Together these results suggest that both PKA and ERK1 kinases are likely regulators of NF-E2 SUMOylation.

## Discussion

The p45/p18 heterodimer (NF-E2) has been reported to be modified by SUMO1 which lead to enhanced transcriptional activity of p45/NF-E2. Also NF-E2-DNA complex formation is increased by cAMP-dependent PKA [7], [9]. However, the inter-relationship between phosphorylation and SUMOylation of NF-E2 is unclear. Our results indicate that PKA enhances the SUMOylation of both p45 and p18 subunits of the p45/p18 heterodimer. Previous reports have also shown that the Ras-Raf-MAP kinase signaling cascade enhanced NF-E2 transcriptional activity [8]. Our *in vitro* results show that ERK1 kinase, apart from PKA, is also able to enhance SUMOylation of the p45 and p18 subunits of the p45/p18 heterodimer, suggesting that ERK1 also regulates NF-E2 SUMOylation. PKA and ERK1 kinases could regulate NF-E2 SUMOylation directly by phosphorylating NF-E2. Many SUMO substrates have a highly conserved motif containing a SUMO consensus site and a proline-directed phosphorylation site (ΨKxExxSP), implying possible phosphorylation-dependent SUMO modification (PDSM) [18]. However, there have been several reports showing that cross talk between phosphorylation and SUMOylation of substrates does not rely on such a motif [26], [27]. Importantly, p45 contains a Ser-169 PKA recognition site and a Lys-368 SUMOylation site. It should be noted that we cannot rule out the possibility that PKA and ERK kinases may regulate NF-E2 SUMOylation indirectly through phosphorylating the SUMO machinery components, leading to an overall stimulation of the SUMOylation activity.

Our work shows that PKA phosphorylation enhances SUMOylation of p45 only when p45 forms a heterodimer with p18, but not when it is in its monomeric form. This result could suggest that PKA-simulated SUMOylation of p45/NF-E2 may not depend on the PDSM motif. However, it is equally likely that both SUMOylation and phosphorylation sites of p45 are brought into proximity (or proper conformation) for functional interaction as a result of the binding of p45 and p18.

Ubiquitously expressed small Maf protein p18 was shown to be modified by SUMO2/3 when in a homodimeric form, leading to transcriptional repression [15]. Yet, it is unclear if the physical properties of p18 affect its SUMOylation activity. Our findings show that SUMOylation activity of p18 is observable both with and without p45, suggesting that p18 SUMOylation is heterodimer-independent. However, it is unclear whether the monomeric or homodimeric form of p18 facilitates its SUMOylation activity. Nevertheless, it is clear that heterodimerization of p18 with p45 drives transcriptional activation of NF-E2, whereas the p18 homodimer induces transcriptional repression. Thus, an interacting balance between the p18 and its heterodimeric partner p45 may serve as a molecular switch for gene expression. Our results therefore suggest that heterodimer formation of p45 with p18 can serve as a physical switch for post-translational modifications, notably phosphorylation and SUMOylation, and thus regulate the transcriptional activity NF-E2.

## Materials and Methods

### Plasmid Constructs

Plasmid DNA encoding full-length p45 and p18 were subcloned into the baculovirus expression vector pFastBac HTb (Invitrogen) and subsequently transposed into recombinant bacmid using the Bac-to-Bac system (Invitrogen). PCR with commercial primers (Invitrogen) confirmed the presence of the p45 and p18 bacmids. DNA encoding full-length p18 was subcloned into the glutathione S-transferase (GST) fusion protein *Escherichia coli* expression vector, pGEX-KG [28]. The resulting plasmids are designated pGEX-p18. For construction of the plasmid expressing pGEX-p18K14R, the Lys-14 within wild-type pGEX-p18 was mutated to arginine by using the QuickChange site-directed mutagenesis kit (Stratagene). Plasmids encoding amino acids 1–97 of SUMO1 (pQE30-SUMO1^(1–97)^), full-length Ubc9 (pQE30-Ubc9), full-length SAE1 and full-length SAE2 have been described previously [29].

### Protein Expression

For the baculoviral expression of the p45/p18 heterodimer, p45- and p18-containing bacmid constructs were co-transfected into SF21 insect cells for P1 baculoviral stock preparation. The recombinant baculovirus of p45 and p18 were then co-infected into SF21 insect cells in 15-cm plates for 7–8 days to amplify P2 and P3 baculoviral stock. Optimal ratios of p45 and p18 P3 virus stock were co-infected in SF21 insect cells for 72 hours at 27°C. The cells were collected and stored at −70°C. Baculoviral expression of SAE1/SAE2 enzyme was described previously [29].

For protein expression of GST-p18, GST-p18K14R, His_6_-SUMO1 and His_6_-Ubc9, plasmids pGEX-p18, pGEX-p18K14R, pQE30-SUMO1^(1–97)^ and pQE30-Ubc9 were transformed into the *E. coli* TOP10 strain. The resulting transformants were cultured in LB medium containing ampicillin (50 µg/ml) with shaking at 37°C. Isopropyl 1-thio-ß-D-galactopyranoside (IPTG) was added to a final concentration of 0.4 mM when *E. coli* cell growth reached an OD_600_ value of 0.3, and the cultures were grown for an extra 3 hours at 37°C. The cells were then collected and stored at −70°C.

### Protein Purification

The insect cells co-infected with p45 and p18 were lysed in insect cell lysis buffer [1% NP40, 350 mM NaCl, l50 mM Tris (pH 8.0), 1 mM DTT, 1 mM PMSF]. The cell lysates were centrifuged and the supernatant were filtered through a 0.45 µm filter. The filtered protein supernatant was injected into the automated FPLC (Fast Performance Liquid Chromatography) instrument supplied with a Mono Q sepharose anion-exchange column (GE Healthcare). The flow rate was maintained at 0.5 ml/min at 4°C. The proteins were eluted by an increasing gradient of NaCl. All fractions were initially resolved with SDS-PAGE and immunoblot. Fractions containing p45/p18 heterodimer were further purified with Ni-NTA affinity column (Qiagen). The purified p45/p18 heterodimer was resolved by 12.5% SDS-PAGE followed by Coomassie blue staining and immunoblot. All immunoblots were probed with anti-p45 and anti-p18 antibodies. The purification of baculoviral-expressed SAE1/His_6_-SAE2 was described previously [29].

*E. coli* cells expressing His_6_-SUMO1 and His_6_-Ubc9 were resuspended in binding buffer [20 mM sodium phosphate (pH 7.9), 100 mM NaCl, 1% Triton X-100, 1 mM PMSF] for sonication and were subjected to Ni-NTA affinity purification (Qiagen). *E. coli* cells expressing GST-tagged p18 and p18K14R were resuspended in 1× PBS buffer (140 mM NaCl, 2.7 mM KCl, 10 mM Na_2_HPO_4_, 1.8 mM KH_2_PO_4_, pH 7.3) containing 1 mM PMSF and 1 mg/ml lysozyme and sonicated. Cell lysates were then centrifuged and supernatants were purified on a glutathione sepharose 4B column (1 ml bed volume). GST-p18 and GST-p18K14R were individually eluted with glutathione elution buffer [50 mM Tris-HCl (pH 7.4) and 10 mM reduced glutathione (pH 8.0)]. The purified His_6_-SUMO1, His_6_-Ubc9, GST-p18 and GST-p18K14R were resolved by 12.5% and 15% SDS-PAGE, followed by detection with anti-SUMO1, anti-Ubc9, and anti-GST antibodies, respectively.

### *In vitro* SUMOylation

To determine whether SUMOylation of p45/NF-E2 is regulated by phosphorylation, various concentrations (8 nM, 15 nM, 30 nM and 60 nM) of PKA or (9 nM, 18 nM and 35 nM) of ERK1 were mixed with His_6_-SUMO1 (1 µg), His_6_-Ubc9 (1 µg), SAE1/His_6_-SAE2 (2.25 ng/µl), substrate protein [p45/p18 heterodimer or p45 alone (1 µg)], ATP (5 mM) and SUMO reaction buffer (20 mM HEPES, pH 7.5; 5 mM MgCl_2_) in a total reaction volume of 20 µl. All reactions were incubated for 60 min at 37°C. Samples were then resolved by 11.25% and 12.5% SDS-PAGE and proteins detected with anti-p45 and anti-p18 antibodies.

To determine whether SUMOylation of p18 is affected by PKA, GST-p18 or GST-p18K14R was added to the SUMOylation mixture containing SUMO machinery components with increasing concentrations (12 nM and 30 nM) of PKA at 37°C for 60 min. The reactions were subsequently resolved by 10% SDS-PAGE followed by immunoblotting with an anti-GST antibody. Various concentration (20 µM, 60 µM and 100 µM) of PKA peptide inhibitor (Promega) was added to SUMOylation mixture containing SUMO machinery components, GST-p18 and 30 nM PKA at 37°C for 60 min. The reactions were subsequently resolved by 10% SDS-PAGE followed by immunoblotting with anti-GST antibody.
